# Biotechnology: Overcoming biological barriers to nucleic acid delivery using lipid nanoparticles

**DOI:** 10.1371/journal.pbio.3002105

**Published:** 2023-04-24

**Authors:** Alex G. Hamilton, Kelsey L. Swingle, Michael J. Mitchell

**Affiliations:** 1 Department of Bioengineering, University of Pennsylvania, Philadelphia, Pennsylvania, United States of America; 2 Abramson Cancer Center, Perelman School of Medicine, University of Pennsylvania, Philadelphia, Pennsylvania, United States of America; 3 Institute for Immunology, Perelman School of Medicine, University of Pennsylvania, Philadelphia, Pennsylvania, United States of America; 4 Cardiovascular Institute, Perelman School of Medicine, University of Pennsylvania, Philadelphia, Pennsylvania, United States of America; 5 Institute for Regenerative Medicine, Perelman School of Medicine, University of Pennsylvania, Philadelphia, Pennsylvania, United States of America; 6 Penn Institute for RNA Innovation, Perelman School of Medicine, University of Pennsylvania, Philadelphia, Pennsylvania, United States of America

## Abstract

The promise of therapeutic nucleic acids has long been tempered by difficulty in overcoming biological barriers to their delivery. The past two decades have seen the development of ionizable lipid nanoparticles as a vehicle for nucleic acid delivery and their translation to the clinic.

## Abstract 2

Over the past 20 years, lipid-based nanoparticles have been engineered to overcome the biological barriers to nucleic acid delivery. This Perspective looks at their development for use in vaccines, immunotherapies, protein replacement therapies, and gene therapies.

This article is part of the *PLOS Biology* 20th Anniversary Collection.

Nucleic acids have garnered great interest in recent years as therapeutic cargoes due to their broad potential to precisely modulate the transcriptome, including the ability to affect targets previously considered “undruggable” [1]. This potential has begun to be realized through clinical advancements such as Onpattro, an FDA-approved siRNA-based therapeutic for hereditary transthyretin-mediated amyloidosis (hATTR); the FDA-approved Pfizer/BioNTech and Moderna COVID-19 vaccines; and the gene editing therapeutic NTLA-2001 for hATTR amyloidosis, currently in clinical trials [1,2].

Numerous classes of nucleic acid cargo have been explored for therapeutic use, including both DNAs and RNAs (Fig 1A), but nucleic acid-based therapies face several extracellular obstacles to their successful delivery (Fig 1B). For *in vivo* applications, the first such obstacle is localization to the site of interest; as treatments are often systemically administered, the therapeutic must first reach the region of interest through circulation (biodistribution) and exit systemic circulation to perform its local function (extravasation) [3]. Nucleic acid-based therapeutics in systemic circulation also must circumvent serum nucleases, which readily degrade their cargo. Innate immune responses compose another barrier against nucleic acid therapeutics: adsorption of serum opsonins can lead to phagocytic clearance [3,4]. Finally, even if the therapeutic can surmount these barriers to reach the cell, it still needs to make its way inside the cell, which is made difficult by the relatively large size of nucleic acids and their negative charge under physiological conditions, complicating transport across the cell membrane.

**Fig 1:**
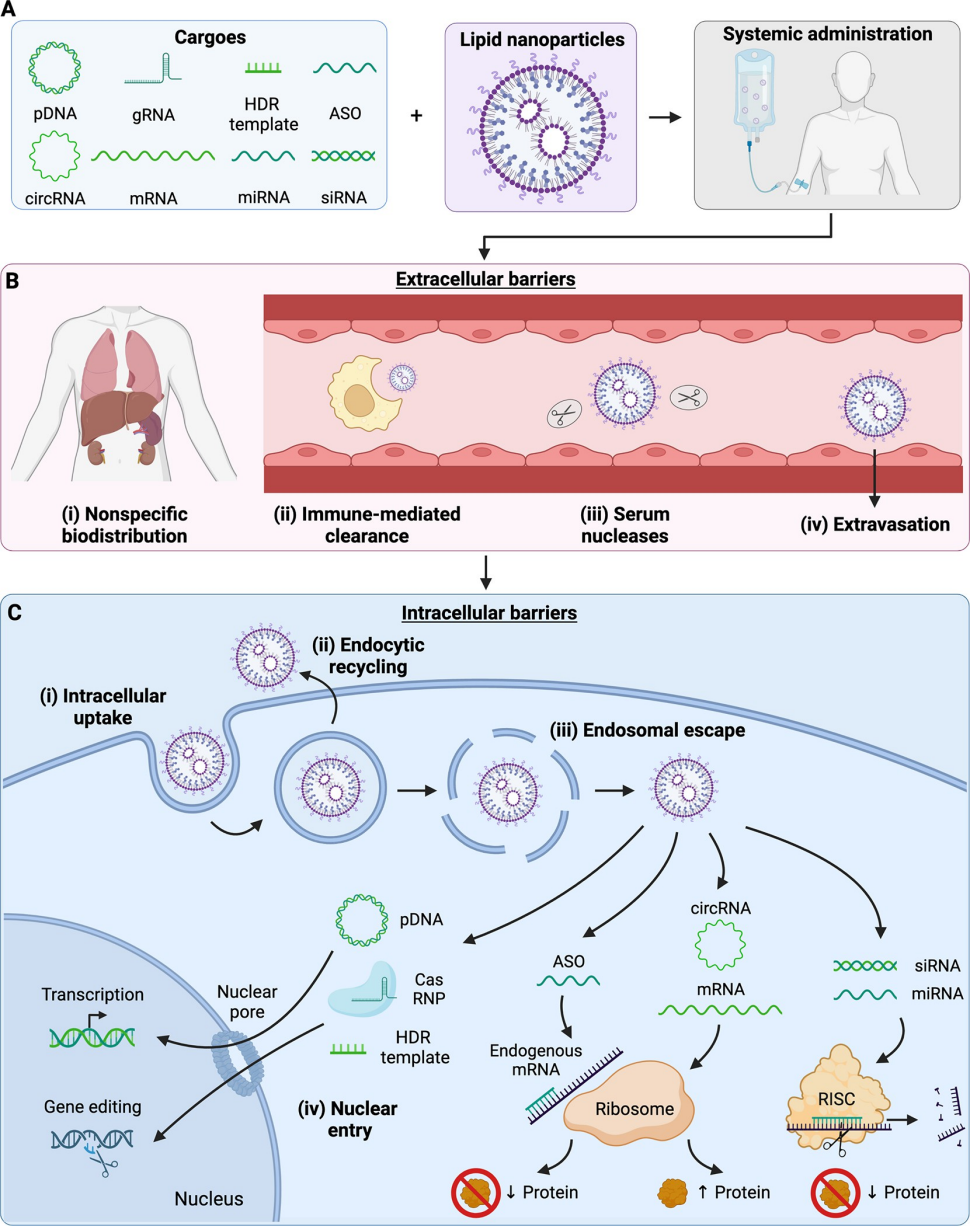
Extracellular and intracellular barriers to nucleic acid delivery. **A.** Lipid nanoparticles are versatile drug carriers and can be employed to encapsulate diverse nucleic acid cargoes, including plasmid DNA (pDNA), messenger RNA (mRNA), and circular RNA (circRNA) for gene delivery; antisense oligomers (ASOs), microRNA (miRNA), and small interfering RNA (siRNA) for gene silencing; and guide RNA (gRNA), mRNA, and homology-directed repair (HDR) templates for gene editing applications. **B.** Following systemic administration, nucleic acid therapeutics face extracellular barriers including nonspecific biodistribution, clearance by phagocytic immune cells, cargo degradation by serum nucleases, and extravasation across endothelial barriers. **C.** Upon reaching the cell, nucleic acid therapeutics face intracellular barriers, the first of these being intracellular uptake. Following successful uptake into endosomes, nucleic acid therapeutics must avoid endocytic recycling to stay within the cell and escape the endolysosomal pathway to reach the cytoplasm. Once inside the cytoplasm, siRNA and miRNA cargoes can associate with the RNA-induced silencing complex (RISC) to recognize and cleave endogenous mRNA, reducing target protein production. Similarly, ASOs in the cytoplasm can bind to complementary sequences within endogenous mRNA, resulting in reduced protein expression through mechanisms including steric hindrance at the ribosome. Assuming proper structure and chemical composition, exogenous mRNA and circRNA can be translated at the ribosome to produce the encoded protein of interest. Other cargoes require nuclear translocation to accomplish their function. pDNA requires nuclear entry to enable its transcription to mRNA and subsequent translation. CRISPR-associated (Cas) ribonucleoplexes (RNPs) consisting of gRNA and translated Cas protein must enter the nucleus to access genomic DNA and perform their gene editing function. In the case of gene knock-in using HDR, DNA templates must also make their way into the nucleus. Created with BioRender.com.

Once inside the cell, nucleic acid-based therapies face further barriers to functionality that are just as onerous as those found in the extracellular environment (Fig 1C). The degree and nature of these obstacles depend on the nucleic acid’s target destination within the cell, whether that be the cytoplasm or the nucleus. A major intracellular barrier to delivery of these cargoes is the endolysosomal pathway, as nucleic acid-based therapeutics commonly rely on endosomal uptake for cellular delivery [3,4]. Endosomal compartmentalization makes these carriers vulnerable to endocytic recycling, through which the carrier is eliminated from the cell. If the endosome does stay within the cell, as it matures, it is acidified and merged with nuclease-rich lysosomes to digest the endosomal contents. Nucleic acid therapeutics must find a way to escape this pathway with their cargo intact. For those cargoes which must reach the nucleus, the nucleic acid must further traffic across the nuclear membrane.

Several classes of lipid carrier have been developed to circumvent the above biological barriers to therapeutic nucleic acid delivery. These lipid-based carriers, in broad strokes, package hydrophilic and delicate nucleic acid cargo within a hydrophobic lipid exterior to confer protection, enhance stability and circulation time, and facilitate transport across hydrophobic biological membranes. The progenitor of these lipid carriers is the liposome, a membrane bilayer system well known for encapsulating small molecules that was first used for nucleic delivery in 1978 [5]. Over a decade later, in 1989, these liposome carriers were modified with permanently charged cationic lipids (lipidoids) to facilitate cargo complexation and endosomal escape, but these cationic lipids were clinically limited due to toxicity [4,5]. To overcome these toxicity-related issues, an engineered, pH-responsive (“ionizable”) lipidoid component was introduced to the formulation to establish a new class of lipid-based carrier—ionizable lipid nanoparticles (LNPs)—as a platform for nucleic acid delivery in 2005 [5]. This ionizable lipidoid was an early ancestor of the DLin-MC3-DMA ionizable lipidoid used in Alnylam Pharmaceuticals’ Onpattro, which became the first FDA-approved RNA interference therapeutic in 2018 [5,6].

One of the hallmarks of LNPs is their active approach to endosomal escape: their ionizable lipidoid component becomes charged upon endosomal acidification, disrupting the endosomal membrane and releasing cargo into the cell [6]. This endows LNPs with the ability to potently deliver nucleic acids into the cytoplasm and beyond. The design and synthesis of lipidoids with enhanced potency and reduced toxicity is a major focus of current LNP research, but intellectual property considerations around these lipidoids present an additional barrier to clinical translation [7]. Advances in rapid synthesis methods and high-throughput screening techniques will be needed to establish robust structure-activity relationships for the large libraries of lipidoids currently being developed pre-clinically [8].

Beyond ionizable lipidoid structure, surface chemistry, lipid composition, and administration route are also key determinants of LNP fate. LNPs are typically formulated with components containing hydrophilic, bulky moieties such as lipid-anchored poly(ethylene glycol) (PEG) to reduce immune recognition and prolong circulation [6]. In the past few years, much attention has been given to additional surface functionalization schemes, including the use of PEG alternatives to avoid hypersensitivity reactions and the introduction of active targeting moieties such as antibodies and peptides to assist in delivery to specific cells and tissues [6,9]. Lipid composition also has a role in achieving tissue-specific nucleic acid delivery with LNPs. Upon intravenous administration, traditional LNP formulations deliver their cargo predominantly to the liver [10]. Extrahepatic delivery of nucleic acid cargo through the development of novel lipid excipients for LNP formulation is an active area of research in the LNP field; achieving extrahepatic LNP-mediated nucleic acid delivery will be critical for the clinical application of this carrier beyond liver-centric diseases [10,11]. Besides intravenous administration, alternative injection routes including intramuscular administration and intranasal administration offer the potential for potent extrahepatic nucleic acid delivery. Moderna and Pfizer/BioNTech’s COVID-19 vaccines, first given emergency use authorization in 2020, rely upon intramuscular injection of mRNA LNPs and currently represent the most clinically advanced non-viral platform for nucleic acid delivery.

As the state of the art in lipid-based nucleic acid carriers has advanced and the field’s understanding of them has grown, interest in nucleic acid therapeutics has renewed. Coupled with the clinical successes of nucleic acid delivery platforms, this has resulted in broad investigation into therapies as varied as infectious disease vaccines, cancer immunotherapies, and treatments for congenital disorders such as cystic fibrosis [6]. Across these clinical applications, most therapies have relied on “classical” nucleic acids such as mRNA and siRNA, but interest is growing in the delivery of novel nucleic acid such as circRNA and gene editing cargoes (Fig 1A) for more sustained or even permanent gene expression for vaccine and protein replacement applications. Forays are also being made into broadening understanding of lipid chemistry, advancing the field’s knowledge of protein corona formation on LNPs and its influences on tissue tropism, which will be invaluable in designing new LNPs for extrahepatic delivery [10,12]. While some aspects of LNP behavior remain a “black box”, such as endosomal escape, we remain confident that mechanistic studies in the coming years will shed light on these phenomena and enable a renaissance in lipid carrier design. The future is bright for nucleic acid therapies and the lipid carriers, such as LNPs, that make them possible.
